# The Effect of Root, Shoot and Seed Extracts of The Iranian Thymus L. (Family: Lamiaceae) Species on HIV-1 Replication and CD4 Expression 

**DOI:** 10.22074/cellj.2016.4321

**Published:** 2016-05-30

**Authors:** Maryam Soleimani Farsani, Mandana Behbahani, Hamid Zarkesh Isfahani

**Affiliations:** 1 Department of Biotechnology, Faculty of Advanced Sciences and Technologies, University of Isfahan, Isfahan, Iran; 2 Department of Immunology, Medical Sciences Faculty, Isfahan University of Medical Sciences, Isfahan, Iran

**Keywords:** Root, Flow-Cytometer, HIV-1, Shoot, Thymus

## Abstract

**Objective:**

The genus Thymus L. is a cushion plant that was previously used for the treatment of bronchitis and rheumatism. The present investigation was carried out to study the effects of root, shoot, leaf and seed extracts of five Thymus species and subspecies on peripheral blood mononuclear cells (PBMCs) toxicity and HIV-1 replication.

**Materials and Methods:**

In this experimental study, the activity of the Thymus extracts on HIV-1 replication and lymphocytes population were examined respectively using HIV-1 p24 Antigen kit and flow-cytometer. The Thymus species effect was investigated, including Thymus kotschyanus, Thymus vulgaris, Thymus carmanicus, Thymus daenensis subspecies lancifolius and Thymus daenensis subspecies daenensis.

**Results:**

The effect of root methanol extracts of all species on PBMCs proliferation was significantly higher than the other extracts. The intensity of CD4, CD3 and CD45 were decreased in the presence of all root extracts. Although the average median fluorescence intensity (MFI) values of CD19 were increased in the cells treated with these extracts. All methanol extracts showed anti-HIV-1 activity at high concentrations (200 and 500 µg/ml). Anti-HIV-1 activity of Thymus daenensis subspecies daenensis was significantly more than the other species.

**Conclusion:**

These results demonstrated that root extracts of Thymus species might be a good candidate to investigate anti-HIV infection *in vivo*.

## Introduction

HIV-1 is one of the most common infectious diseases, causing acquired immunodeficiency syndrome (AIDS). The current anti-HIV-1 drugs have many disadvantages including resistance, toxicity and high prices. Within the recent decades, many efforts have been carried out to find natural products with anti-HIV-1 activity (1,2). The genus Thymus (Lamiaceae) is a cushion plant which is also classified as perennial herb (3). It is an aromatic plant with a native compatibility to Mediterranean region. It is known as "Avishan" or "Azorbeh" in Persian. Approximately, 400 species of Thymus have been reported throughout the world (4). Among them, 14 species are introduced from Iran. As a case, according to Morales (5) a new species, named Thymus (Th.) marandensis Jamzad, has been added to the others growing in Iran. Thymus vulgaris L., as one of the most frequent Thymus species, is currently utilized in cosmetic, food and pharmaceutical industries. In addition, several investigations have been performed to highlight the composition and biological effects of other genus species including Th. daenensis Celak, Th. migricus Klokov and Des., Th. pubescens Boiss (6,7). The essential oils and extracts of Thymus species are widely used in pharmaceuticals, cosmetics and perfume industries as well as food products (8,9). Studies demonstrated that the Thymus species could have anti-inflammatory, anti-viral, anti-fungal, anti-bacterial, anti-oxidant and anti-cancer effects (10,13). Recently, several bioactive compounds have boon isolated from Thymus species including thymol, carvacrol, borneol, p-cymene, γ-terpinene, tanen (14,15). The extracts of some Thymus species (e.g. Th. Maroccanus Ball, Th. zygis L., Th. pallidus Coss., Th. leptobotrys Murb, Th. algeriensis Boiss. Reut. and Th. broussonetii Boiss.) have been reported to induce a stimulating effect on lymphocyte proliferation (8,16). However, there is no scientific study available about anti-HIV-1 activity of the Iranian Thymus species. The present study focused on the latter objective to evaluate the effect of methanol extracts of the Iranian Thymus species on HIV-1 replication and lymphocytes population. 

## Materials and Methods

This experimental study was approved by Faculty of Advanced Sciences and Technologies at University of Isfahan. 

### Plant material 

Thymus species and subspecies (Th. kotschyanus Boiss, Th. vulgaris, Th. carmanicus Jalas, Th. daenensis subsp. lancifolius and Th. daenensis subsp. daenensis) were investigated in this study (Table 1). 

### Preparation of extracts

The samples were separated into flower, leaf, stem and root parts. The plant material was dried in shadow and then was powdered. Methanol extract (98%) of dried and powdered samples were prepared. The extraction was performed thrice at 40˚C. The solvent was filtered and evaporated in a vacuum rotary evaporator (Steroglass, Italy) at 45˚C. The residue was placed in a freeze dryer (Zirbus, Germany). 

### Cells and viruses

Human healthy donors’ blood samples were collected in heparinized tubes. Peripheral blood mononuclear cells (PBMCs) were isolated by Lymphodex density centrifugation. The cells were cultured in Roswell Park Memorial Institute (RPMI) media supplemented with 15% fetal bovine serum (FBS), penicillin solution (100 µg/ml), streptomycin (100 µg/ml) and L-Glutamin (2 mM). All of the reagents were purchased from Gibco (Germany). The PB-MCs were incubated at 37˚C and 5% CO_2_ condition (Biotek, South Korea). A virus stock of HIV-1 subtype B was obtained from Alzahra Hospital (Isfahan, Iran). The virus titers were measured using HIV-1 p24 antigen kit (BioMerieux, France). The viruses were stored at -70˚C until use.

**Table 1:** List of the Thymus species and their localities


Taxa	Localities

*Th. kotschyanus Boiss*	Soleimani 15372, 1570 m, Isfahan, Iran (herbarium of Isfahan University)
*Th. daenensis subspecies lancifolius Celak*	Soleimani 15373, Najafabad 1649, Isfahan, Iran (herbarium of Isfahan University)
*Th. daenensis subspecies daenensis Celak*	Soleimani 15374, Najafabad 1649, Isfahan, Iran (herbarium of Isfahan University)
*Th. vulgaris L.*	Soleimani 15375, 1570 m, Isfahan, Iran (herbarium of Isfahan University)
*Th. carmanicus*	Jalas Soleimani 15376, 1570 m, Isfahan, Iran (herbarium of Isfahan University)


### Human peripheral blood mononuclear cells cytotoxicity assay

The cellular toxicity of PBMCs was estimated in the presence of the different extracts of Thymus species using MTT assay (17). For that, The methanol extracts at concentrations of 10, 100, 200, 800 and 1600 µg/ml were added to 180 µl of cell suspension (6×10^5^cell per well) and incubated for 72 hours in 37˚C and 5% CO_2_ . After three days of culture, 20 µl of MTT (0.5 mg/ ml) was added to each well and the mixture was incubated for two hours at the same condition. Then 50 μl of PrOH/HCl/TX (0.04 M HCL/2propanol/10% triton 100x) was separately added to all wells and incubated for six hours. Optical density of the cells was measured at 570 nm by micro-plate spectrophotometer (Stat fax 2100, Awareness Technology Inc., USA). Each concentration was tested three times. In addition, dimethyl sulfoxide (DMSO) was used as a negative control in this experiment. The 50% cytotoxic concentration (CC_50_) of all pure compounds was also calculated. All experiments were carried out in triplicate. 

### Anti-viral activity

Anti-HIV-1 activity of root extracts of all Thymus species was studied via the HIV-1 p24 Antigen kit according to our previous study (18). This kit is used to measure the amounts of HIV-1 Gag p24 antigen in cell culture medium. The protocol was followed as described by the manufacturer. Briefly, 6×10^5^PBMCs were infected with 0.5 multiplicity of infection (MOI) for HIV-1 subtype A, in 500 µl medium supplemented with different concentrations of extract (200 and 500 µg/ml) and incubated at 37˚C for 12 hours. The infected cells were then washed and overlaid with medium at different concentrations of extract. 0.1% DMSO and two concentrations of Zidovudine (AZT, 5 and 10 µg/ml) were also used as negative and positive controls, respectively. After three days of incubation, the overlay medium was collected to quantify the HIV-1 p24 core protein. Finally, the overlay medium was transferred to the coated 96-well plate for the p24 assay. Optical density of virus was measured at 450 nm by micro-plate spectrophotometer. A selectivity index (SI) was calculated for each viral strain by the ratio of CC50 to 50% of anti-viral effective concentration (EC_50_). 

### Analysis of CD4, CD3, CD45 and CD19 expressions by flow-cytometer

The percentage values of CD4, CD3, CD45 and CD19 lymphocytes and their expression intensities on PBMCs, in the presence of Thymus root extracts, were evaluated by flow-cytometer (FACScan, USA). In this experiment, 5×10^6^ PBMCs were cultured in 24-well plates. After 72 hours of incubation at 37˚C, the cells were washed with PBS. These cells were then separately incubated with the saturating concentration of PE anti-human CD4, RPE CY5 anti-human CD3, FITC anti-human CD45 and FITC anti-human CD19 monoclonal antibodies (Cyto Matin Gene, Iran) for 20 minutes at 4˚C. Lymphocytes were gated, accorde ing to their forward and side scattered properties. At least, 10,000 events were acquired for each sample. Each incubation was followed by two washing steps. Data acquisition was achieved using BD Cell Quest software. 

## Results

### Analysis of human peripheral blood mononuclear cells cytotoxicity assay

**Fig.1:**
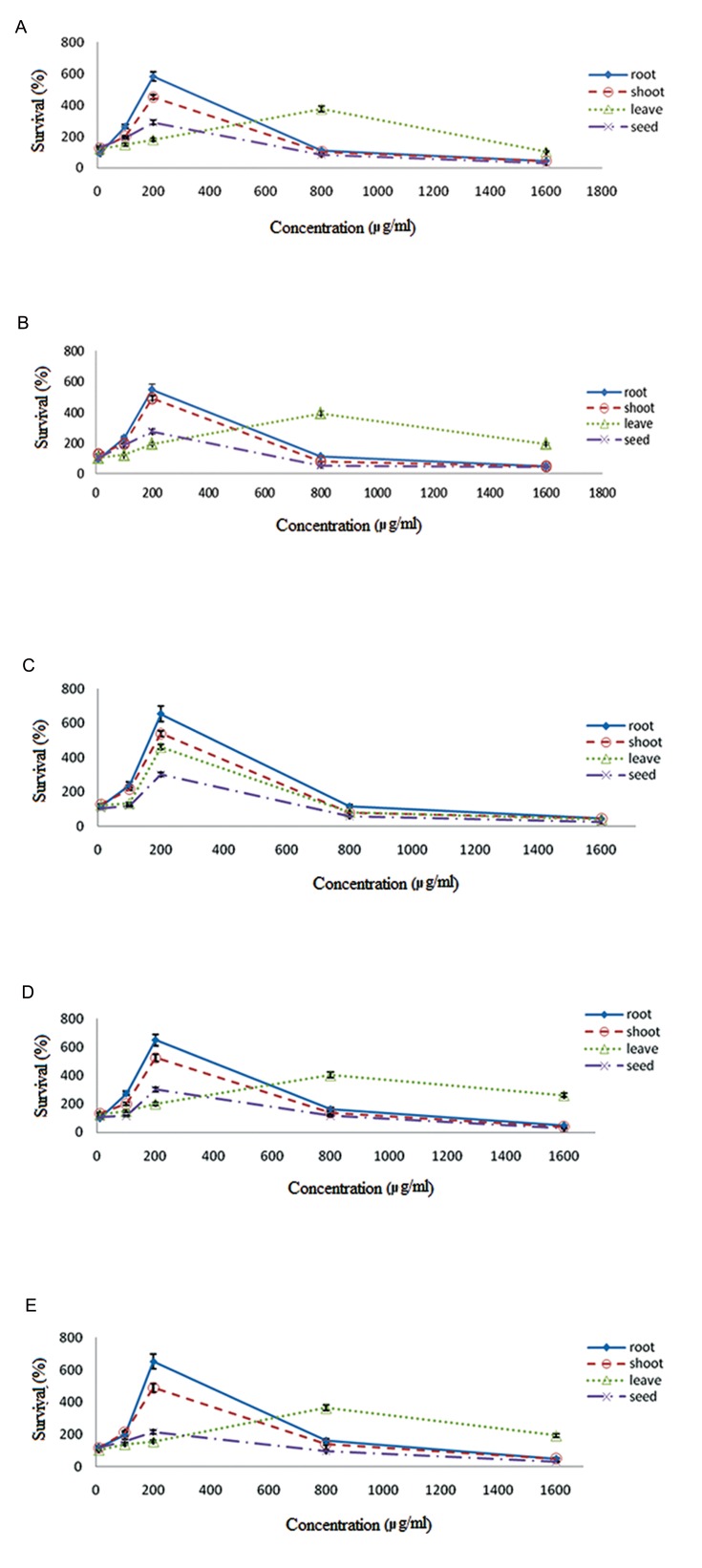
The effects of the methanol extracts obtained from different parts of Thymus species on PBMCs number in different concentrations: A. Th. kotschyanus, B. Th .daenensis subspecies lancifolius, C. Th. Vulgaris, D. Th . daenensis subspecies daenensis and E. Th. Carmanicus. The axes of X and Y demonstrates survival (%) and concentration (µg/ml), respectively. PBMSCs; Peripheral blood mononuclear cells.

Cellular toxicity effect of methanol extracts, obtained from different parts of Thymus species, on PBMCs was investigated. The results demonstrated that methanol extract increases PBMC numbers in a dose dependent manner (Fig.1). The root extracts increased PBMC numbers more than the other parts of plant. Thus, the PBMC numbers was increased five-six fold. The highest effects of shoot, root, leaf and seed extracts of Th. Carmanicus, Th. kotschyanus, Th. daenensis subspecies daenensis and Th. daenensis subspecies lancifolius on PBMC numbers were obtained at the extract concentrations of 200, 200, 200 and 800 µg/ml, respectively (Fig.1A-E). 

The highest effect of all parts of Th. vulgaris on PBMC numbers was obtained at 200 µg/ml (Fig.1C). The results showed that cell CC_50_ values for all of the mentioned species are more than 1000. Seed extracts showed the minimum CC_50_ values in the species. The CC_50_ values of root extracts in Th. kotschyanus, Th. carmanicus, Th. vulgaris, Th. daenensis subspecies lancifolius and Th. daenensis subspecies daenensis were obtained at the concentrations of 1590, 1570, 1500, 1580 and 1590 µg/ml, respectively. The extracts with the highest activity on PBMC numbers were selected for evaluation of anti-HIV-1 activity and CD4 expression. 

### Anti-viral activity of root extracts in Thymus species

**Fig.2:**
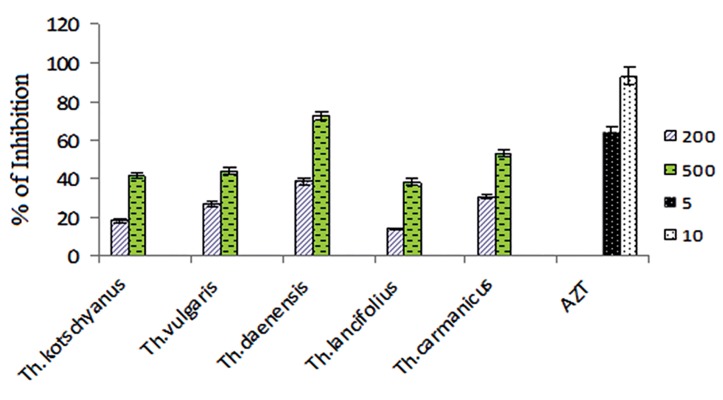
The effects of root extracts of Thymus species (200 and 500 µg/ml) and AZT (5 and 10 µg/ml) on HIV-1 replication in PBMCs. The IC_50_ of each extract was calculated using regression line. Each bar represents the mean of SD of three independent experiments. AZT; Zidovudine, PBMSCs; Peripheral blood mononuclear cells and IC_50_ ; 50% inhibitory concentration.

The root extracts of all *Thymus* species exhibited an anti-viral activity at the concentrations of 200 and 500 µg/ml (Fig.2). Findings showed that the extract of *Th. daenensis* subspecies *daenensis* inhibited HIV-1 replication with an EC_50_ value of 300 µg/ml. The EC_50_ of other extracts were more than 500 µg/ml. EC_50_ values for all species were more than standard (AZT). The calculated SI were obtained <3.18, <3.11, <3.00, <3.16, 5.26 for *Th. kotschyanus, Th. carmanicus, Th. vulgaris, Th. daenensis* subspecies *lancifolius* and *Th. daenensis* subspecies *daenensis*, respectively. 

### The effect of root extracts on CD4, CD3, CD45 and CD19 expressions

**Fig.3:**
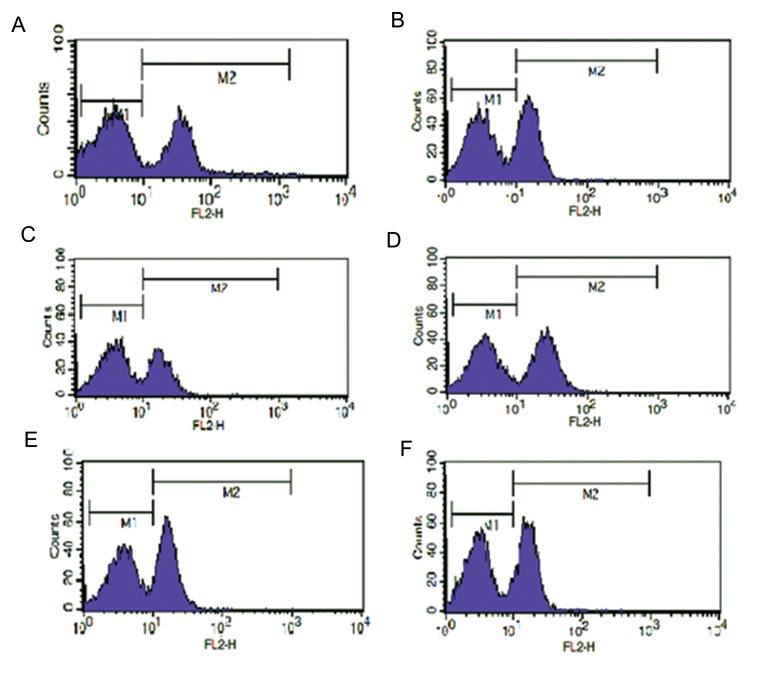
The effects of DMSO and root extracts on the expression of CD4+ T cells in PBMCs. Data in each plot represent 10,000 events for cells stained with PE conjugated monoclonal antibody specific to human CD4. Histograms show the fluorescent intensity (X-axis) versus cell number (Y-axis). A. CD4 expression of T cells in PBMCs treated with DMSO, B. CD4 expression of T cells in PBMCs treated with root extract of *Th. Kotschyanus, C. Th. Vulgaris, D. Th. daenensis* subspecies *daenensis, E. Th. daenensis* subspecies *lancifolius* and *F. Th. Carmanicus*. DMSO; Dimethyl sulfoxide and PBMSCs; Peripheral blood mononuclear cells.

The effect of the *Thymus* species root extracts on the frequency and average of mean fluorescent intensity (MFI) of CD4+ T cells in PBMCs have been summarized in the table 2. The results showed that methanol root extracts of all mentioned species did not have any effect on the frequency of CD4+ T cells in PBMCs. However, the average MFI value of this entire cell population, while it was treated with all root extracts, were significantly reduced to a ratio of 40-60% compared to the control. A marked shift in the CD4+ T cell population to the left was observed in cells treated with *Th. kotschyanus, Th. daenensis* subspecies *lancifolius, Th. carmanicus, Th. vulgaris* and *Th. daenensis* subspecies *daenensis* extracts, with a resulting MFI value of 25.72, 24.41, 24.24, 22.72 and 15.62, respectively (Fig.3). 

Observations also demonstrated that these five extracts did not have any effect on the frequency of CD3+ T cells, CD19+ and CD45+ lymphocytes, however, the average MFI values of these markers have been changed in the cells treated with extracts. The MFI values were also reduced to 3580% for CD3 and 20-60% for CD45 lymphocytes compared to controls (Table 1). Although, the average MFI value of CD+19 lymphocytes was increased for the cells treated with the root extracts of *Th. daenensis* (subspecies daenensis and subspecies *lancifolius*) and *Th. carmanicus* to 40%, compared to the control (Table 2). 

**Table 2:** CD4, CD3, CD45 and CD19 expression levels on human lymphocytes after treatment with root extracts of the Thymus species


Sample	CD3+ T cells		CD45+ cells		CD19+ B cells		CD4+ T cells	
	Frequency (%)	MFI	Frequency (%)	MFI	Frequency (%)	MFI	Frequency (%)	MFI

Control (treated with DMSO)	61.53	264.2	88.27	207.45	3.74	14.81	37.18	41.84
Th. kotschyanus	63.1	65.71	88	153.24	2.86	15.25	41.32	16.12
Th. vulgaris	59.21	15.52	86.71	85	4.53	15.13	37.57	19.12
Th. daenensis subspecies Daenensis	63.24	83.8	90.33	161.12	2.64	18.42	43.43	26.22
Th. daenensis subspecies Lancifolius	60	57.86	86.46	144.68	2.73	24	43.26	17.43
Th. carmanicus	62.63	78.4	88.1	119	3.61	22.43	42.96	17.6


MFI; Median fluorescence intensity and DMSO; Dimethyl sulfoxide.

## Discussion

The present study demonstrated that methanol extracts of all parts of *Th. kotschyanus, Th. carmanicus, Th. vulgaris, Th. daenensis* subspecies *lancifolius* and *Th. daenensis* subspecies *daenensis* do not have any cytotoxicity on PBMCs. The results of this research are compatible with some of the previous studies performed on several *Thymus* species, including *Th. broussonettii, Th. marroccanus, Th. zygis, Th. pallidus, Th. leptobotrys* and *Th. algeriensis* (8,17). It has been determined that these species not only have no cytotoxicity on PBMCs, but also could increase lymphocyte proliferation in a dose dependent manner (8,17,19,20). Our results also demonstrated that root methanol extracts increased PBMC numbers more than the other parts of plant, while the results of Layne et al. (21) showed that the root and shoot extracts of *Th. vulgaris* is very rich in respect to essential oil especially carvacrol. The proliferation effect of carvacrol isolated from *Thymus* species has been reported previously (8,21). It should be pointed out that increasing effect of the root extracts of the studied species on PBMCs is related to its carvacrol and other essential oils. The present results also demonstrated that the root extracts of the species decreased both MFI values of CD4+ T cells in PBMCs and HIV-1 replication. MFI value of CD3+ T and CD45+ cells were also decreased in cells treated with root methanol extracts. Regarding that multimeric CD4 binding is mandatory for efficient HIV-1 infection (22), CD4 receptor density must play an essential role in the efficiency of viral infectivity (20). Thus, drugs with CD4 down-regulatory activity can inhibit virus entry by reducing the CD4 receptor density that is required for infection (23). As a novel finding, we determined the anti-HIV-1 activity of the presented species. Recently, several groups of natural product with anti-HIV-1 property have been detected which act on a range of processes of HIV life cycle such as entry, integration and maturation (24). The anti-reverse transcriptase activity of *Th. quinquecostatus* Celakovsky and *Th. serpyllum L.* has been reported previously (25). Therefore, the root extracts of these *Thymus* species and subspecies might prevent HIV-1 reverse transcriptase enzyme on the early stage of HIV infection. Nevertheless, further studies are needed to verify the mechanism of these extracts. 

## Conclusion

We demonstrated that root extracts of *Thymus* species are able to prevent HIV-1 replication, through reducing the CD4 receptor density. 
